# Have Standard Tests of Cognitive Function Been Misappropriated in the Study of Cognitive Enhancement?

**DOI:** 10.3389/fnhum.2017.00276

**Published:** 2017-05-24

**Authors:** Iseult A. Cremen, Richard G. Carson

**Affiliations:** ^1^Trinity College Institute of Neuroscience and School of Psychology, Trinity College DublinDublin, Ireland; ^2^School of Psychology, Queen’s University BelfastBelfast, United Kingdom

**Keywords:** aging, physical activity, coordination training, motor fitness, brain imaging

## Abstract

In the past decade, there has emerged a vast research literature dealing with attempts to harness brain plasticity in older adults, with a view to improving cognitive function. Since cognitive training (CT) has shown restricted utility in this regard, attention has increasingly turned to interventions that use adjunct procedures such as motor training or physical activity (PA). As evidence builds that these have some efficacy, it becomes necessary to ensure that the outcome measures being used to infer causal influence upon cognitive function are subjected to appropriate critical appraisal. It has been highlighted previously that the choice of specific tasks used to demonstrate transfer to the cognitive domain is of critical importance. In the context of most intervention studies, standardized tests and batteries of cognitive function are de rigueur. The argument presented here is that the latent constructs to which these tests relate are not usually subject to a sufficient level of analytic scrutiny. We present the historical origins of some exemplar tests, and give particular consideration to the limits on explanatory scope that are implied by their composition and the nature of their deployment. In addition to surveying the validity of these tests when used to appraise intervention-related changes in cognitive function, we also consider their neurophysiological correlates. In particular, we argue that the broadly distributed brain activity associated with the performance of many tests of cognitive function, extending to the classical motor networks, permits the impact of interventions based on motor training or PA to be better understood.

## Introduction

A compelling body of evidence indicates that the aging brain’s structure and function can be altered by factors amenable to intervention in later life, such as physical activity (PA) and social enhancement (for review see Ballesteros et al., 2015). As a result, the research literature now documents a multitude of attempts to harness the brain’s capability for adaptive reorganization and change i.e., “neuroplasticity”. The majority of these endeavors aim to improve “cognition”. It is readily apparent that this term encompasses a wide range of putative capabilities. Expressed in the language of cognitive science, these may include: executive function, memory, attention and processing speed. In the context of many intervention studies, standardized tests and batteries are employed to operationalize these elements and examine the degree to which they are amenable to directed change. The argument presented herein is that the latent constructs to which such tests relate are not usually subject to a sufficient level of analytic scrutiny. Relatedly, empirical evidence to the effect that a specific intervention has an impact upon a particular measure of cognitive function does not necessarily lend support in terms relevant to how an older adult functions in daily life (e.g., Simons et al., 2016). Ecological validity is frequently defined as the extent to which results obtained in controlled experimental settings apply to real-world naturalistic settings (Tupper and Cicerone, 1990). In order for interventions to be deemed truly effective therefore, the benefits should generalize to functions germane to everyday life, such as competence and autonomy, and not simply the specific tasks upon which one is trained or tested (Lövdén et al., 2010). With respect to many of the tests that are employed to evaluate interventions to improve cognition, ecological validity is bound by the limits on explanatory scope that are implied by their composition (as distinct from their customary interpretation). This limitation notwithstanding, with the widespread availability of neuroimaging, it is further becoming apparent that the neurophysiological correlates of test performance are frequently at odds with those that are assumed by their adherents. Beyond highlighting the challenges posed by these considerations, we examine how they permit the seemingly positive impact of some forms of PA upon tests of cognitive function to be better understood.

## Seeking to Improve Cognitive Function in Older Adults

Of the approaches that have been applied in an effort to improve cognitive function in older adults, the most common are cognitive training (CT) and PA. The former typically encompasses games or exercises designed to challenge specific cognitive skills. In contrast, PA interventions use exercise and movement program to improve physical capability, with the expectation that there will also be a positive impact in the cognitive domain (Bamidis et al., 2014). In relation to CT, although meta-analytic reviews have shown small improvements on measures of intermediate transfer of training gains to untrained tasks, there is little evidence of transfer to “real world” cognitive skills (Lampit et al., 2014; Melby-Lervåg et al., 2016; Simons et al., 2016). As Druin Burch noted: “Doing something repeatedly can make you better at it, which is not the same as saying it makes you better” (Burch, 2014, p.2).

PA interventions, in the forms of aerobic exercise and resistance training, appear to yield somewhat more consistent positive effects upon cognitive function in older adults (Colcombe and Kramer, 2003; Smith et al., 2010). Classes of PA that place greater explicit emphasis upon the generation of coordinated movement (and allude to a concept of “motor fitness”) are now receiving particular attention (e.g., Voelcker-Rehage et al., 2011; Forte et al., 2013; Berryman et al., 2014; Moreau et al., 2015; Johann et al., 2016). In some cases the rationale for such approaches includes an emphasis on the “cognitive” demands of coordinated goal-directed movement, such as anticipatory planning and mapping sensation to action (Voelcker-Rehage et al., 2010). As the boundaries between variants of “cognitive” and “physical” training become blurred, it is an opportune moment to consider critically the nature of outcome measures used to infer causal influence upon cognitive function.

Necessarily the choice of task(s) used to demonstrate transfer of training related adaptations to the cognitive domain is of critical importance, as it determines the weight of the inferences that can be drawn. In view of this dependency, it has been recommended that multiple measures should be used to minimize measurement error and provide reliable and accurate estimates of the target construct (e.g., Shipstead et al., 2012; Moreau et al., 2016). In many cases, standardized tests and batteries (e.g., CAMDEX, Cogstate, The NIH Toolbox Cognition Battery) are de rigueur. Such selections are designed to ensure that the measurement instruments have been validated, are widely used and accepted, and permit comparisons across multiple studies. In the majority of cases however, these tests were devised to achieve an aim radically distinct from that of measuring enhancements in the cognitive functioning of older adults. We offer a perspective that includes an historical dimension, a delineation of limits on inference, and is informed by contemporary developments in neuroimaging. In examining the ecological and construct validity of prototypical tests, prudence necessarily emerges in relation to that which may be construed from their use (Heinrichs, 1990; Franzen and Arnett, 1997; Chaytor and Schmitter-Edgecombe, 2003).

## Some Tests of Cognition Function

We do not seek to be comprehensive with regard to the tests of cognitive function that are employed in contemporary cognitive neurorehabilitation. Instead, we discuss a small number of exemplar tests, not in an attempt to target their specific uses and/or misappropriation, but rather to highlight the limits on their explanatory scope. In addition, we draw attention to the fact that the broadly distributed brain activity associated with the performance of these tests precludes reification in terms of any discrete cognitive processes (Uttal, 2013). Indeed, the most pervasive feature of the brain activation associated with these tests is engagement of the classical motor networks. We focus on common tests used to assess three “core executive functions” (Diamond, 2013, p.135): inhibition/inhibitory control, Working memory (WM) and cognitive flexibility/set-shifting (Miyake et al., 2000; Diamond, 2013).

### Response Inhibition Tasks

Response inhibition tasks are used commonly to assess a facility to suppress prepotent actions and carry out a goal-directed response (Diamond, 2013). Response inhibition is said to be a key factor in successful cognitive and motor control (Chambers et al., 2009). The Eriksen Flanker task—perhaps the most common variant, was devised by Eriksen and Eriksen (1974). It is a speeded response time task that explores the effect of “flanker” distractor stimuli on target identification reaction time (RT). RT typically increases when the target stimulus is surrounded by “incongruent” distractor stimuli—letters or shapes from the target set that require a different response. The Flanker task is included in the NIH Toolbox Cognition Battery (Gershon et al., 2013), the Attentional Network Test (ANT; Fan et al., 2002) and a variant forms part of the CANTAB battery (Attention Switching Task). It is emblematic of a class of response inhibition tests, including the Simon task, that have been used to assess a supposed ability to suppress responses that are inappropriate in a particular context.

The Flanker task appears frequently within the cognitive enhancement literature, in particular in studies exploring the associations between cognitive function and PA (e.g., Colcombe et al., 2004; Davranche et al., 2009). This footing may in itself allude to the neural processes and adaptations to which the task may be sensitive. In Colcombe et al. (2004), a 6-month aerobic exercise intervention was shown to enhance cognition in older adults—as evidenced by improvement on the Eriksen Flanker task. It is no surprise then, that this test has since been used in many other studies examining aerobic PA (e.g., McMorris et al., 2009; Weng et al., 2015), resistance training (e.g., Liu-Ambrose et al., 2012), yoga (Gothe et al., 2013) and recently in those focusing on motor fitness (e.g., Voelcker-Rehage et al., 2010; Schoene et al., 2015). It has been included as a measure of executive function; described variously as a test of selective attention, response inhibition and information processing. In light of the fundamental characteristics of the test however, and in view of the nature of the interventions that give rise to a change in the level of performance—i.e., having an emphasis upon the selection of voluntary movements, the conclusion might be drawn that it will have a high degree of sensitivity to the functional state of elements within the classical motor networks.

The prototypical design is that in which responses in the presence of congruent or incongruent flankers are each compared to responses to neutral flankers. In many studies a direct comparison is made between responses in the presence of congruent flankers and responses in the presence of incongruent flankers. In the context of both designs, the interpretation of outcomes relies upon a “subtraction logic”, whereby it is assumed that the same motor output is required in each instance, and that any difference between conditions (expressed via any given dependent measure) derives from other sources. With respect to the flanker task, the resulting contrast measure is hypothesized to be a “pure” measure of response inhibition, divorced of motor influence.

In a series of brain imaging studies, all of which employed the subtraction logic, it has been shown that during performance of the Flanker task, various elements of the cortical motor network including the pre-supplementary motor areas (pre-SMA) and SMAs (Bunge et al., 2002; Taylor et al., 2007) and Brodmann area 6 (BA6) more broadly defined (Zurawska Vel Grajewska et al., 2011; Caruana et al., 2014) exhibit differential activity in the congruent and incongruent conditions. The characteristics of neural activity registered in primary motor areas also differ reliably in the context of responses made in the presence of congruent and incongruent flankers (Grent-’t-Jong et al., 2013; see also Praamstra et al., 1998, 1999; Verleger et al., 2009). On the basis of such evidence it has been proposed that the executive control nominally sampled by these tests represents an evolutionary extension of the frontal cortex-basal ganglia loops that guide resolution of (motor) response conflict, such that the role of the supporting neural mechanisms extends to a range of processes including the reorienting of attention and the updating of WM (Neubert et al., 2013).

In line with the more general argument that is advanced in this piece, it should also be noted that, while at a phenomenological level the same motor response (e.g., depression of a response key) may appear to be generated in each condition, the state of the “motor circuitry” varies systematically across conditions. This can be revealed in a number of ways. As the latency between the onset of electromyographic (EMG) activity and the start of the response movement is longer when incongruent flankers are present than when congruent flankers are present (Coles et al., 1985; Eriksen et al., 1985; Smid et al., 1990), and shorter in the congruent condition than the neutral condition (Smid et al., 1990), differences in muscle activation dynamics are implied. It can also be shown that the time course of changes in the excitability of corticospinal projections to motoneurons innervating muscles that act as an agonists in generating the manual response when incongruent flankers are present, is distinct from that associated with responses made in the presence of congruent flankers (and in control conditions; Michelet et al., 2010; see also Klein et al., 2014; Duque et al., 2016).

In the absence of additional measurements, the possibility that intervention related changes in the state of motor networks contribute to changes in the magnitude of flanker effects cannot be excluded. Thus, when drawing inferences on the basis of the flanker task, and indeed response inhibition tests more generally, it is necessary to recognize that motor function is central to their interpretation.

### Working Memory Tasks

WM is frequently described in such terms as the ability to hold and manipulate information in one’s mind (Baddeley and Hitch, 1994; Smith and Jonides, 1999). The *n*-back task was introduced by Kirchner (1958) to measure differences in performance between younger and older adults on a “paced” task. A light flashed on and off in sequence at one of 12 locations, and participants were required to press buttons indicating where the light had gone out* n* positions before. As *n* increased, older adults (60–84) performed more poorly on this task when compared to younger adults (18–24). This was attributed to a slowing down of “central organizing processes of the brain” (p.357). A commonly overlooked element of this original experiment was that when the time allowed for the response was increased (from 1.5 s to 4.5 s), the performance of older adults improved substantially, leading the authors to conclude “the time factor plays an important part in the results” (p. 356).

In modern variants, stimuli may be auditory, visual, or in the case of the more recently developed dual *n-*back, auditory and verbal stimuli may be presented simultaneously (Jaeggi et al., 2003). The latter version has become popular as a WM training task (Jaeggi et al., 2008). In spite of weak convergence with other measures of WM (e.g., poor correlation with operational span (OSPAN), Kane et al., 2007, and with backward digit span, Miller et al., 2009), the task has become paradigmatic in both clinical and experimental settings. It is included in popular neuropsychological test batteries (e.g., Cogstate; the Penn Computerized Neurocognitive Battery, Gur et al., 2010), and has been used as a measure of WM in a number of physical intervention studies that have sought to enhance cognitive function (e.g., Kramer et al., 2002; Hansen et al., 2004; Gothe et al., 2013). It is however rarely the case that steps are taken in an attempt to parse separately the component elements of task performance.

A strong case can be made that when the *n*-back task is used in the context of intervention studies, subtraction logic should be applied. This is borne of the recognition that the *n*-back is a dual-task with two dissociable subcomponents. These comprise a WM updating subtask—involving the “encoding, manipulation, search and selection of information in WM”, and a matching subtask—requiring the comparison of a currently presented stimulus with the (previous) one already selected (Watter et al., 2001, p. 999). In most implementations of the paradigm, participants are afforded sufficient time to complete the selection of the *n*-back stimulus prior to the presentation of a new stimulus, and thus the demands of the matching subtask are in principle the same across different* n*-back variants (i.e., 0-back, 1-back, 2- back etc). Generally this characteristic simplifies the interpretation of the data derived from the *n-back* paradigm (i.e., in relation to the impact of variations in “memory load”). In the case of changes in performance arising from an intervention however, it is not possible to exclude the possibility that a decrease in RT (or increase in accuracy) obtained for a single variant (e.g., 2-back) is attributable to a change in execution of the matching subtask. To take account of this caveat, it is necessary to express the level of performance achieved in variants with presumed higher memory load (e.g., 2-back) relative to a reference condition that also includes the matching sub-task (e.g., 0-back). We are aware of very few intervention studies with a focus on motor training or PA in which this step has been taken. Indeed, in many of the studies which have reported a positive impact upon *n*-back performance, either a single *n* was included (e.g., Hansen et al., 2004; Stroth et al., 2010; Hogan et al., 2013), or in cases in which data for more than one *n* were available (e.g., Erickson et al., 2013), normalization procedures were not applied. It is notable that with only one exception of which we are aware (Weng et al., 2015), with respect to those studies in which performance measures for more than one level of *n* were included in the analysis design, differential effects (e.g., 0-back vs. 2-back) of a motor training or PA intervention have not been reported (e.g., Kramer et al., 2002; Gothe et al., 2013). In the absence of suitable contrasts or normalization procedures, it is not evident that intervention-related improvements in the performance of an *n*-back task variant can be attributed to changes in the efficiency of WM processes. It also remains to be determined whether different *n-back* variants are characterized by distinct motor signatures—in the manner of those that distinguish the various conditions of the flanker task.

Although the speeded response selection characteristics of the matching subtask, that is intrinsic to the *n*-back, make plain that significant demands are imposed on the motor system, the ramifications of this are also amenable to scrutiny in terms of neurophysiology. In two activation likelihood estimation (ALE) meta-analyses published simultaneously (Glahn et al., 2005; Owen et al., 2005), 12 brain atlas delineated areas of activation were highlighted in association with performance of the *n*-back task. In the subset of only five areas for which there was a corresponding response across the two analyses BA6 was prominent. Although it is one of the largest regions in the Brodmann scheme, and a diversity of functions would thus be anticipated, since area 6 includes premotor cortex and SMA it necessarily has a fundamental role in regulating motor output. With respect to WM tests however, engagement of the cortical motor network is not simply a unique feature of the *n-back* protocol. In a comparison of seven further meta-analyses (in addition to the two that used the *n*-back procedure), Uttal (2013) noted that forty-seven Brodmann areas were reported as being activated during WM tasks (i.e., across the nine meta-analyses). Of these 47 brain regions, only BA6 was designated as being activated in every case. Indeed, in the context of extremely large variations in regional brain activation, the detection of signal in cortical motor areas during WM tasks is one of the most robust findings (e.g., Niendam et al., 2012). It has furthermore been determined that the threshold at which motor responses to transcranial magnetic stimulation (TMS) can be obtained—which is a measure of the excitability of corticospinal projections from primary motor cortex (M1), is negatively correlated (across individuals) with performance in *n*-back tasks (Schicktanz et al., 2013; Bridgman et al., 2016). As with response inhibition tests therefore, intervention related improvements in the performance of the *n*-back test in particular, and of WM tasks in general, may be mediated, at least in part, by adaptive changes within parts of the cortical motor network.

### Cognitive Flexibility Tests

The third “core” element of executive function is considered to be cognitive flexibility/set shifting. The Trail Making Test (TMT) is used frequently in this context (Butler et al., 1991; Sellers and Nadler, 1993; Rabin et al., 2005). It is variously described as measuring cognitive flexibility, processing speed, sequencing, (Arbuthnott and Frank, 2000; Bowie and Harvey, 2006; Ashendorf et al., 2013), visual search, scanning and executive functions (Tombaugh, 2004). The TMT, originally devised in 1938 and known first as “Distributed Attention” and then as Partington’s “Pathway Test” (Partington and Leiter, 1949), was originally intended as a test of speed, eye-hand coordination, alertness and distributed attention. In the 1940s, its inclusion in both the Army Individual Test Battery and the Halstead-Reitan Neuropsychological Battery (HRNB, Reitan and Wolfson, 1985) ensured its continued use and propagation in a wide range of research settings. It is now included in many national longitudinal studies of aging (e.g., the Harvard Aging Brain Study, The Irish Longitudinal Study of Aging (TILDA), The Aging, Demographics and Memory Study (ADAMS), etc.).

In the first part of the test (TMT-A), lines are drawn sequentially in order to connect 25 encircled numbers distributed on a sheet of paper. In the second part (TMT-B), the requirements are similar, with the exception that the individual being tested must alternate between letters and numbers, in increasing numerical and alphabetical order (e.g., 1, A, 2, B, 3, C, etc.). Performance is expressed in terms of the time taken to complete separately each portion of the test. On the basis of associations obtained between individual scores for elements of the TMT and other psychometric tests, it has been surmised that the TMT-A draws mainly upon visuo-perceptual abilities, that the TMT-B is primarily an expression of WM and task-switching ability (Sánchez-Cubillo et al., 2009, p.448). As it has been assumed that the demands in relation to “motor speed and visual scanning” are equivalent for both parts, further scores are also often derived (Arbuthnott and Frank, 2000, p.519). Most frequently these are the TMT-B—TMT-A difference score, and the TMT-B/TMT-A ratio. It is believed that by reflecting the additional requirements of the TMT-B, these scores measure “executive control” (p.519). In view of the fact that in the prototypical variants of the task, the distance between the targets is greater in the TMT-B than the TMT-A (Gaudino et al., 1995; Bowie and Harvey, 2006), a ratio score provides the more appropriate form of normalization. Indeed, it has been argued that it is essential to employ the ratio score when the goal is to evaluate executive function (Oosterman et al., 2010).

It is perhaps surprising therefore that many of the studies that have reported a positive impact of motor training or PA have treated the TMT-A and TMT-B separately (or singly), or reported a single additive measure (Emery et al., 1998; Scherder et al., 2005; Baker et al., 2010; Nguyen and Kruse, 2012; Vaughan et al., 2014; Eggenberger et al., 2015; Tazkari, 2016; de Natale et al., 2017; Gregory et al., 2017; Jonasson et al., 2017). Notwithstanding any evidence that completion times for either part of the TMT may be correlated (across individuals) with other measures of cognitive function (e.g., Sánchez-Cubillo et al., 2009), an intervention-induced change in the performance of TMT-A or TMT-B cannot simply be attributed to an effect on a specific faculty such as cognitive flexibility or set shifting. Contingent variations in the influence of other factors that mediate the successful completion of these tasks must also be contemplated.

On a prima facie basis, the intrinsic nature of the TMT is such as to suggest that individual levels of performance will be particularly sensitive to integrity of motor function. Indeed, this much was implied in its original formulation. The neurophysiological evidence is consistent with this supposition. When variants of the TMT specifically adapted for neuroimaging are employed, for example using a “virtual stylus” (Zakzanis et al., 2005) or button box (Jacobson et al., 2011) adapted to collect responses within the confines of an fMRI scanner, the contrast between the TMT-A and TMT-B reveals differences in BOLD response in the dorsal part of M1 (Jacobson et al., 2011; see also Zakzanis et al., 2005; Kodabashi et al., 2014). With respect to a clinical population, it has been reported that the TMT-B—TMT-A difference score is correlated with upper arm central motor conduction delay (Ravaglia et al., 2002). Using the TMT-B, Allen et al. (2011) registered the presence of task-related activation in the left precentral gyrus (M1), bilateral premotor cortex and the medial pre-SMA (see also Moll et al., 2002; Horacek et al., 2006). When measured using functional near-infrared spectroscopy, bilateral activity has been detected during the TMT-B task in premotor regions (Müller et al., 2014). In a further study using the same imaging procedures, activity was also detected in the M1, with older participants exhibiting greater task related changes in O_2_Hb signal strength in this brain region, and in the right premotor cortex during both variants (Hagen et al., 2014). Employing EEG-derived measures, Wölwer et al. (2012) reported associations between TMT-B task performance and the current density of M1 designated sources. In light of the evidence of cortical motor network mediation, and particularly in view of the limitations that are associated with measures derived from a single TMT variant, the explanation that intervention associated enhancements in performance of the TMT-A or TMT-B (i.e., considered separately or additively) arise from adaptations in motor function, seems most parsimonious.

In a small number of published studies focusing on motor training or PA, a TMT-B/TMT-A ratio score has been employed (Klusmann et al., 2010; Yin et al., 2014; cf, Schoene et al., 2015). In none of these cases was an effect of a motor training or PA intervention demonstrated. Null findings have also been reported in all of the studies known to us in which a TMT-B—TMT-A difference score has been used as a dependent measure (Nagamatsu et al., 2012; Forte et al., 2013; Liu-Ambrose et al., 2016; Barban et al., 2017; de Natale et al., 2017; Jonasson et al., 2017).

Given the evidence that is available presently therefore, there is no basis upon which to suppose that motor training or PA has a reliable impact upon the facets of executive function to which the TMT is thought to be specifically sensitive. While reductions in completion times have been reported for individual task variants (TMT-A or TMT-B), it is not possible to exclude the possibility that these are attributable to changes in some aspects of motor function arising from the particular mode of intervention.

## Conclusions

In the cognitive sciences, when faced with the practical challenges of operationalizing a theoretical construct, the pragmatic turn is to develop an experimental paradigm to capture its key features. Subsequently however, idiosyncratic features of the methodology may become reified as the phenomenon of interest (Nosek et al., 2012)—this may be quite distinct from the construct the paradigm was developed to capture, or indeed is capable of capturing. The point is that an improvement in the performance of a test arising from therapeutic intervention does not entail that the change may be interpreted in terms of the particular facet of cognition assigned by the practitioner to the test. Thus while enhanced performance of the Eriksen Flanker task might be ascribed to an improvement in selective attention, in the absence of convergent evidence, the effect of the intervention can with equal legitimacy be attributed to adaptations related to motor function. Indeed, with respect to many interventions that are based on PA, the latter is the more parsimonious account.

As a research tradition has evolved to explore notionally different aspects of cognition (“WM”, “numeracy”, “executive function” and so on), measured using different paradigms, there has also emerged a tendency to treat mediating brain processes as similarly dissociable in terms of constituent functions. Thus, in an era in which brain imaging has become the tour de force of cognitive neuroscience, and with access to tools that assign activity to specific brain regions, it remains customary to discuss variations in task-dependent activation in terms of the functional localization of various aspects of cognition (Ross, 2010). Necessarily however, the roles in cognition assumed by spatially circumscribed regions of the brain are highly diverse (Anderson, 2014). This much is certainly true of many elements of the cortical motor network. Beyond the specific examples given above, there is overwhelming evidence that their engagement is an obligatory feature of cognition in general, and the performance of tests of cognitive function in particular. In circumstances—such as therapeutic interventions based on PA, in which the purpose is more effective and/or efficient motor output, functional adaptations within motor networks are anticipated. In light of the tests that are employed, there is every reason to believe that many improvements in cognition ascribed to these interventions are also accountable in these terms.

We should strive to ensure that cognitive enhancement remains, at its core, an effort to improve the quality of life for older adults, targeting functional independence and activities of daily life. “Far” transfer from physical training to test-derived measures of cognition offers promise, however the transfer may not be as “far” as is assumed, or as “far” as is required.

## Author Contributions

The authors together wrote the article, jointly contributed the intellectual content and gave approval to the final version of the article to be published.

## Conflict of Interest Statement

The authors declare that the research was conducted in the absence of any commercial or financial relationships that could be construed as a potential conflict of interest.
